# Supplementary material for: Local trait responses and aquatic microclimates increase projected habitat suitability for field-collected Anopheles stephensi in southern Ethiopia
Source: bioRxiv. 2026 Jul 23:2026.07.21.739549. Preprint. [Version 1] doi: 10.64898/2026.07.21.739549 (PMC13419477; doi:10.64898/2026.07.21.739549)
Supplement: Supplement 1 [file media-1.pdf]

Supplementary Information: Local trait responses and aquatic microclimates increase projected habitat suitability for field-collected *Anopheles stephensi* in southern Ethiopia

Contents

1 Sensitivity analyses for temperature support and projection input **2**

1.1 Sensitivity analysis design 2

1.2 Temperature support of projection inputs 3

2 Restricting projections to 19–34°C **4**

3 Refitting the global comparator within the local temperature window **5**

4 Summary **7**

1 Sensitivity analyses for temperature support and projection input

This supplementary document reports sensitivity analyses used to assess whether projected differences between local and global juvenile trait parameterisations were influenced by temperature support and curve-shape effects. The main analysis compared projections of maximal population growth rate, r_{m}, using two trait parameterisations: *Local*, fitted to juvenile survival and development data from field-collected *Anopheles stephensi* from Hawassa, Ethiopia; and *Global*, fitted to an externally derived comparator combining published juvenile trait data. Projections were evaluated using two matched temperature inputs: logger-measured larval habitat water temperature and ERA5-Land 2 m air temperature.

The local juvenile experiments were conducted at 19, 26, 30, and 34°C. The global comparator contained broader thermal support and produced a more clearly unimodal thermal response, so we tested whether the Local–Global difference in projected r_{m} was driven by extrapolation beyond the local experimental range or by the broader support of the global curve.

1.1 Sensitivity analysis design

We performed two sensitivity analyses. We first quantified the proportion of projection temperatures falling within the local experimental temperature range of 19–34°C. We then repeated habitat-level r_{m} summaries after excluding all temperature observations outside this range. This tested whether Local–Global differences persisted when projection was restricted to temperatures directly supported by the local juvenile experiments. Second, we refitted the global comparator using only global juvenile trait observations within 19–34°C. This restricted global model, denoted *Global local-window*, was projected onto the same temperature inputs and compared with the original *Local* and *Global* projections. This tested whether the broader thermal support of the full global comparator, and its associated curve shape, contributed to lower projected suitability.

1.2 Temperature support of projection inputs

Logger-measured habitat water temperatures were generally better supported by the local experimental range than ERA5-Land 2 m air temperatures (Table S1). Across habitats, 65.4–99.8% of logger-water observations fell within 19–34°C. In contrast, only 25.9–34.9% of ERA5-Land air-temperature observations fell within this range. ERA5-Land projections therefore relied much more heavily on temperatures below the local experimental range.

Table S1: Temperature support of projection inputs relative to the local experimental range of 19–34°C.

Temperature source	Habitat	n_{total}	n_{within}	Proportion within	Minimum	Maximum
Logger habitat water	Pool 1 brick making site	411	392	0.954	18.1	28.4
Logger habitat water	Pool 2 brick making site	410	409	0.998	19.9	34.8
Logger habitat water	Pool at carwash site	410	325	0.793	17.5	29.2
Logger habitat water	Swimming pool	410	268	0.654	16.7	30.8
Logger habitat water	Tank 1	343	286	0.834	18.3	25.4
Logger habitat water	Tank 2	343	273	0.796	17.8	29.4
ERA5-Land 2 m air	Pool 1 brick making site	411	111	0.270	13.3	22.5
ERA5-Land 2 m air	Pool 2 brick making site	410	111	0.271	13.3	22.5
ERA5-Land 2 m air	Pool at carwash site	410	143	0.349	13.9	23.4
ERA5-Land 2 m air	Swimming pool	410	142	0.346	13.7	23.4
ERA5-Land 2 m air	Tank 1	343	89	0.259	13.3	22.5
ERA5-Land 2 m air	Tank 2	343	89	0.259	13.3	22.5

2 Restricting projections to 19–34°C

Restricting projections to the local experimental range did not remove the Local–Global difference (Table S2). For logger-measured water temperatures, the Local–Global difference was similar in the full and restricted analyses. The mean habitat-level Local–Global difference was 0.037 under the full logger-water projection and 0.034 after restricting to 19–34°C. Local projections remained higher than Global projections in all six habitats.

For ERA5-Land 2 m air temperature, restricting to 19–34°C substantially reduced the Local–Global difference. The mean habitat-level Local–Global difference was 0.070 under the full ERA5 projection and 0.039 after restricting to the local experimental range. Thus, the larger ERA5-based Local–Global contrast was partly amplified by low-temperature extrapolation. However, Local projections remained higher than Global projections for every habitat after restriction.

Table S2: Local–Global differences in mean projected r_{m} under the full temperature series and after restricting projection temperatures to 19–34°C.

Temperature source	Habitat	Global	Local	Local–Global	Analysis
Logger habitat water	Pool 1 brick making site	0.166	0.201	0.035	Full series
Logger habitat water	Pool 1 brick making site	0.168	0.202	0.034	Restricted
Logger habitat water	Pool 2 brick making site	0.191	0.213	0.022	Full series
Logger habitat water	Pool 2 brick making site	0.191	0.213	0.022	Restricted
Logger habitat water	Pool at carwash site	0.148	0.192	0.045	Full series
Logger habitat water	Pool at carwash site	0.155	0.196	0.041	Restricted
Logger habitat water	Swimming pool	0.160	0.198	0.037	Full series
Logger habitat water	Swimming pool	0.179	0.207	0.028	Restricted
Logger habitat water	Tank 1	0.157	0.197	0.040	Full series
Logger habitat water	Tank 1	0.165	0.201	0.035	Restricted
Logger habitat water	Tank 2	0.147	0.192	0.045	Full series
Logger habitat water	Tank 2	0.155	0.196	0.041	Restricted
ERA5-Land 2 m air	Pool 1 brick making site	0.085	0.157	0.071	Full series
ERA5-Land 2 m air	Pool 1 brick making site	0.157	0.197	0.040	Restricted
ERA5-Land 2 m air	Pool 2 brick making site	0.085	0.157	0.071	Full series
ERA5-Land 2 m air	Pool 2 brick making site	0.157	0.197	0.040	Restricted
ERA5-Land 2 m air	Pool at carwash site	0.099	0.165	0.066	Full series
ERA5-Land 2 m air	Pool at carwash site	0.163	0.199	0.037	Restricted
ERA5-Land 2 m air	Swimming pool	0.099	0.164	0.066	Full series
ERA5-Land 2 m air	Swimming pool	0.164	0.200	0.036	Restricted
ERA5-Land 2 m air	Tank 1	0.083	0.156	0.072	Full series
ERA5-Land 2 m air	Tank 1	0.157	0.197	0.040	Restricted
ERA5-Land 2 m air	Tank 2	0.083	0.156	0.072	Full series
ERA5-Land 2 m air	Tank 2	0.157	0.197	0.040	Restricted

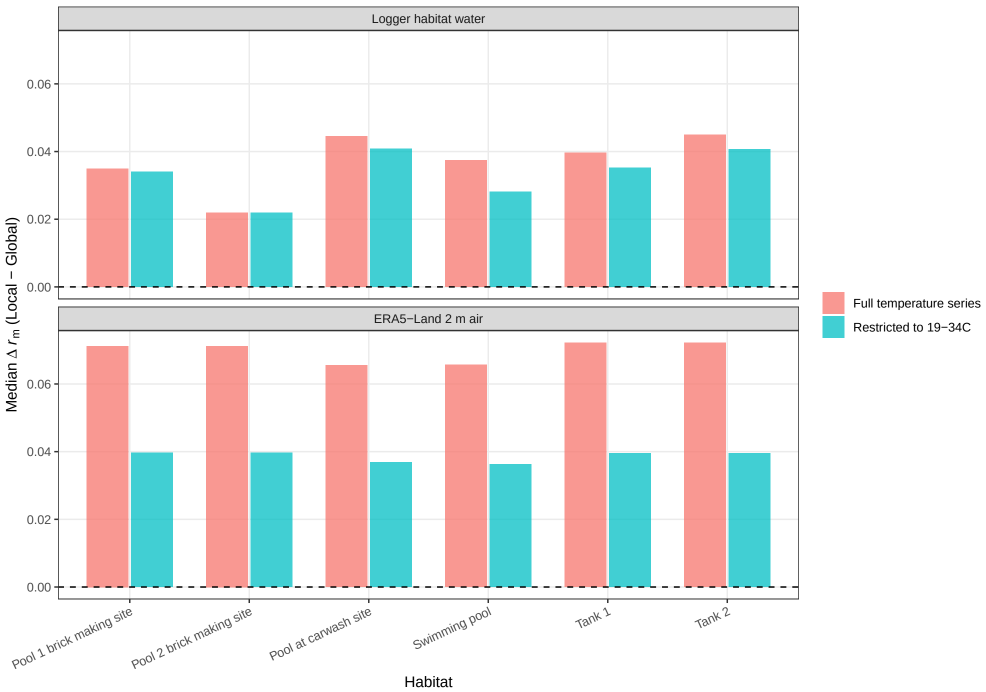

Figure S1: Local–Global difference in mean projected r_{m} under the full temperature series and after restricting projection temperatures to the local experimental range of 19–34°C.

3 Refitting the global comparator within the local temperature window

Restricting the global comparator to observations within 19–34°C produced projections that were similar to the original Global projection for logger-measured water temperatures, but higher than the original Global projection for ERA5-Land air temperatures (Table S3). For logger-measured water temperatures, the restricted global fit changed projected r_{m} only slightly, and the Local projection remained higher in all habitats. For ERA5-Land air temperature, the restricted global fit increased projected r_{m} relative to the original Global comparator, indicating that the broader thermal support and more strongly unimodal shape of the full Global curve contributed to lower projected ERA5 suitability. The restricted global projection nevertheless remained below the Local projection across all habitats.

Table S3: Habitat-level mean projected r_{m} for Local, Global, and Global local-window sensitivity projections. Values are posterior medians and 95% credible intervals.

Temperature source	Habitat	Scenario	Median	Lower	Upper	n_{days}
Logger habitat water	Pool 1 brick making site	Local	0.201	0.194	0.208	18
Logger habitat water	Pool 1 brick making site	Global	0.166	0.163	0.169	18
Logger habitat water	Pool 1 brick making site	Global local-window	0.168	0.164	0.172	18
Logger habitat water	Pool 2 brick making site	Local	0.213	0.206	0.219	18
Logger habitat water	Pool 2 brick making site	Global	0.191	0.188	0.194	18
Logger habitat water	Pool 2 brick making site	Global local-window	0.190	0.187	0.194	18

Continued on next page

Temperature source	Habitat	Scenario	Median	Lower	Upper	n_{days}
Logger habitat water	Pool at carwash site	Local	0.192	0.185	0.199	18
Logger habitat water	Pool at carwash site	Global	0.148	0.145	0.150	18
Logger habitat water	Pool at carwash site	Global local-window	0.152	0.147	0.156	18
Logger habitat water	Swimming pool	Local	0.198	0.191	0.204	18
Logger habitat water	Swimming pool	Global	0.160	0.158	0.163	18
Logger habitat water	Swimming pool	Global local-window	0.164	0.160	0.168	18
Logger habitat water	Tank 1	Local	0.197	0.190	0.203	15
Logger habitat water	Tank 1	Global	0.157	0.154	0.159	15
Logger habitat water	Tank 1	Global local-window	0.160	0.156	0.164	15
Logger habitat water	Tank 2	Local	0.192	0.185	0.198	15
Logger habitat water	Tank 2	Global	0.147	0.144	0.149	15
Logger habitat water	Tank 2	Global local-window	0.151	0.147	0.155	15
ERA5-Land 2 m air	Pool 1 brick making site	Local	0.157	0.147	0.169	18
ERA5-Land 2 m air	Pool 1 brick making site	Global	0.085	0.082	0.088	18
ERA5-Land 2 m air	Pool 1 brick making site	Global local-window	0.100	0.093	0.107	18
ERA5-Land 2 m air	Pool 2 brick making site	Local	0.157	0.147	0.169	18
ERA5-Land 2 m air	Pool 2 brick making site	Global	0.085	0.082	0.088	18
ERA5-Land 2 m air	Pool 2 brick making site	Global local-window	0.100	0.093	0.107	18
ERA5-Land 2 m air	Pool at carwash site	Local	0.165	0.157	0.175	18
ERA5-Land 2 m air	Pool at carwash site	Global	0.099	0.097	0.102	18
ERA5-Land 2 m air	Pool at carwash site	Global local-window	0.112	0.106	0.118	18
ERA5-Land 2 m air	Swimming pool	Local	0.164	0.156	0.175	18
ERA5-Land 2 m air	Swimming pool	Global	0.099	0.096	0.101	18
ERA5-Land 2 m air	Swimming pool	Global local-window	0.111	0.105	0.118	18
ERA5-Land 2 m air	Tank 1	Local	0.156	0.146	0.168	15
ERA5-Land 2 m air	Tank 1	Global	0.083	0.080	0.086	15
ERA5-Land 2 m air	Tank 1	Global local-window	0.098	0.092	0.106	15
ERA5-Land 2 m air	Tank 2	Local	0.156	0.146	0.168	15
ERA5-Land 2 m air	Tank 2	Global	0.083	0.080	0.086	15
ERA5-Land 2 m air	Tank 2	Global local-window	0.098	0.092	0.106	15

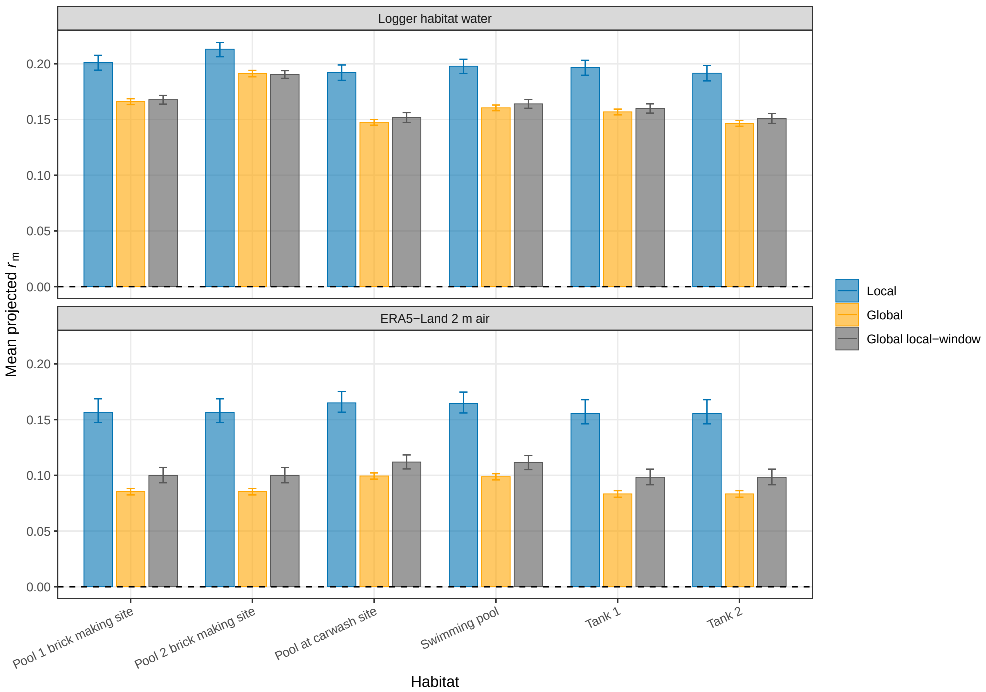

Figure S2: Habitat-level mean projected r_m for the Local, original Global, and restricted Global local-window parameterisations.

4 Summary

The sensitivity analyses qualify but do not overturn the main Local–Global inference. For logger-measured habitat water temperatures, the Local–Global difference was robust to excluding temperatures outside 19–34°C, and the restricted global refit produced projections close to the original Global comparator. This indicates that the main logger-based result is not primarily an extrapolation artefact or a consequence of the global comparator having broader thermal support.

For ERA5-Land 2 m air temperature, most observations fell below the local experimental range, and restricting to 19–34°C substantially reduced the Local–Global difference. The restricted global refit also increased ERA5-based Global projections relative to the original Global comparator. Therefore, the full ERA5-based Local–Global contrast was partly amplified by low-temperature extrapolation and by the more strongly defined lower-temperature limb of the full global curve. Nevertheless, Local projections remained higher than Global projections across all habitats and both temperature inputs under all sensitivity analyses.
